# Role of Hydrophobins in *Aspergillus fumigatus*

**DOI:** 10.3390/jof4010002

**Published:** 2017-12-24

**Authors:** Isabel Valsecchi, Vincent Dupres, Emmanuel Stephen-Victor, J. Iñaki Guijarro, John Gibbons, Rémi Beau, Jagadeesh Bayry, Jean-Yves Coppee, Frank Lafont, Jean-Paul Latgé, Anne Beauvais

**Affiliations:** 1 Aspergillus Unit, Institut Pasteur, 75015 Paris, France; isabel.valsecchi@pasteur.fr (I.V.); remi.beau@pasteur.fr (R.B.); jean-paul.latge@pasteur.fr (J.-P.L.); 2 Unité de RMN des Biomolécules, Institut Pasteur, 75015 Paris, France; inaki.guijarro@pasteur.fr; 3 Centre for Infection and Immunity, Institut Pasteur de Lille-CNRS UMR8204-INSERM U1019-CHRU Lille-Université Lille, 59655 Lille, France; vincent.dupres@ibl.cnrs.fr (V.D.); frank.lafont@cnrs.fr (F.L.); 4 Institut National de la Santé et de la Recherche Médicale, Unité 1138, 75006 Paris, France; esvkai@gmail.com (E.S.-V.); jagadeesh.bayry@crc.jussieu.fr (J.B.); 5 Centre de Recherche des Cordeliers, Université Pierre et Marie Curie–Paris 6, Université Paris Descartes, 75006 Paris, France; 6 Biology Department, Clark University, Worcester, MA 01610, USA; JGibbons@clarku.edu; 7 Transcriptome et Epigénome, Institut Pasteur, 75015 Paris, France; jean-yves.coppee@pasteur.fr

**Keywords:** hydrophobin, rodlet, cell wall, *Aspergillus*

## Abstract

Resistance of *Aspergillus fumigatus* conidia to desiccation and their capacity to reach the alveoli are partly due to the presence of a hydrophobic layer composed of a protein from the hydrophobin family, called RodA, which covers the conidial surface. In *A. fumigatus* there are seven hydrophobins (RodA–RodG) belonging to class I and III. Most of them have never been studied. We constructed single and multiple hydrophobin-deletion mutants until the generation of a hydrophobin-free mutant. The phenotype, immunogenicity, and virulence of the mutants were studied. *RODA* is the most expressed hydrophobin in sporulating cultures, whereas *RODB* is upregulated in biofilm conditions and in vivo Only RodA, however, is responsible for rodlet formation, sporulation, conidial hydrophobicity, resistance to physical insult or anionic dyes, and immunological inertia of the conidia. None of the hydrophobin plays a role in biofilm formation or its hydrophobicity. RodA is the only needed hydrophobin in *A. fumigatus*, conditioning the structure, permeability, hydrophobicity, and immune-inertia of the cell wall surface in conidia. Moreover, the defect of rodlets on the conidial cell wall surface impacts on the drug sensitivity of the fungus.

## 1. Introduction

Hydrophobins are low molecular weight proteins with remarkable physicochemical properties secreted by filamentous fungi [1,2]. Though, in general, hydrophobins show low sequence conservation, these proteins are characterized by their hydrophobicity profiles and an idiosyncratic pattern of eight conserved cysteine residues that form four disulfide linkages. Hydrophobins are secreted in a soluble form that self-associates into amphipathic layers at hydrophobic/hydrophilic or air/water interfaces [1]. The surfactant and amphipathic nature of the hydrophobin layers help in the formation of essential aerial structures of filamentous fungi, such as hyphae, fruiting bodies, and spores [3]. Based on their hydrophobicity pattern, morphology of the monolayers they form and their solubility in detergents, hydrophobins are divided in two classes. Class I hydrophobins form functional amyloid fibers organized in layers with rodlet morphology, while Class II hydrophobin layers show no defined morphology, in general. Recently, an intermediate class of hydrophobins (Class III) has been described [2,4].

*Aspergillus fumigatus* is the most ubiquitous airborne fungal pathogen. The airborne spores (called conidia) are inhaled by the human population and can cause a wide range of diseases, from common allergies to fatal infections [5]. Immunocompromised individuals are especially susceptible to *Aspergillus* colonization, which can culminate in fatal invasive aspergillosis (IA).

Like all fungi, *A. fumigatus* has a protective cell wall that is at the forefront of the interactions with host immune components. The *A. fumigatus* cell wall is mainly composed of different polysaccharides, α-(1,3)-glucan, chitin, galactomannan, β-(1,3)-glucan, and mycelial-specific galactosaminogalactan. In the infective morphotype conidia, the cell wall is covered by a melanin layer and an outer layer with rodlet morphology consisting of amyloid fibers composed of the protein RodA, which belongs to the hydrophobin family. The capacity of airborne conidia to reach alveoli is due to the highly hydrophobic layer of rodlets that facilitates air dispersion [6]. Furthermore, the rodlet layer formed by RodA masks conidial recognition by the human innate immune system [7]. In addition to *RODA*, (AFUA_5G09580; AFUB_057130) (*Aspergillus* Genome Database) identified five more hydrophobin genes in *A. fumigatus*, *RODB* (AFUA_1G175250, AFUB_016640), *RODC* (AFUA_8G07060, AFUB_080740), *RODD* (AFUA_5G01490, AFUB_050030), *RODE* (AFUA_8G05890, AFUB_081650), and *RODF* (AFUA_5G03280, AFUB_051810) [8]. *RODG* (AFUA_2G14661) was identified later, due to mis-annotation in the *A. fumigatus* strain Af293 database [2]. *RODG* is located on the *A. fumigatus* Af1163 scaffold scf_000002, on the opposite strand from an annotated gene (AFUB_030300). A search for new hydrophobins in filamentous fungi using successive blasts on the *Aspergillus* comparative database led to the identification of three more proteins after the sixth blast (AFUA_5G09960, AFUA_7G00970 and AFUA_8G01770) [4]. However, these new proteins were not taken into account herein, because these proteins are predicted as adhesins by the FungalRV adhesin predictor and, hence, they do not show the hallmarks of hydrophobins [8,9]. Sequence analysis in silico placed RodA, RodB, and RodC in Class I, and, thus, as competent to form rodlet layers. RodF and RodG belong to Class III, which contains hydrophobins with intermediate (between classes I and II) or atypical characteristics [2,4]. The allocation of RodD and RodE to the hydrophobin family is controversial [2]. Among all Rod proteins, only RodA is well characterized. RodB was shown to also be present in conidia, but disruption of the *RODB* gene showed that this hydrophobin, although homologous to RodA, was not involved in rodlet formation [6]. Hence, the role of RodB in *A. fumigatus* biology has to be elucidated. RodC to RodG have never been studied. With regard to virulence, Δ*rodA* was shown ex vivo to be less virulent than the wild-type, although this mutant was still able to kill mice given that the rodlet layer normally disappears during germination [6,10,11]. The effects of deleting other hydrophobin genes on virulence of *A. fumigatus* are completely unknown.

In the present study, simple and multiple mutants disrupted in hydrophobins were used to investigate the role of various hydrophobins in *A. fumigatus* biology. As shown here, among all hydrophobins, only RodA was responsible for the hydrophobicity, formation of rodlets, physical resistance, and immunological inertia of the conidia. Moreover, there was no complementation of the lack of one hydrophobin by another one. As Δ*rodA* stimulates host immunity, we evaluated the effects of a mutant disrupted in all *ROD* genes, except *RODA*, on the host immune system.

## 2. Material and Methods

### 2.1. Strains and Culture Conditions

The *A. fumigatus* reference strain used in this study is CEA17ΔakuBKU80 (ku80), deficient in non-homologous end joining [12]. This strain, which originates from the clinical isolate CBS 144-89, is as pathogenic as CBS 144-89 in experimental murine aspergillosis. Strain ku80 was used to generate hydrophobin-deleted mutants. All strains were grown in 2% (*w*/*v*) malt agar slants from which conidia were recovered after three weeks by vortexing with 0.05% (*v*/*v*) Tween 20 aqueous solution (Tw-H_2_O). Minimal medium (MM) [13] was used for the construction of the mutants and antifungal susceptibility; glucose 3%-yeast extract (1%) (GYE) medium for adherence, germination and biofilm tests [14]; and GI-10% fetal calf serum was used for human dendritic cell (DC) culture [15].

The *Pseudomonas aeruginosa* strain used in this study was Pa14, a bacterial strain labeled with the green-fluorescent protein GFP (a kind gift of Niels Høiby, Copenhagen University Hospital, Denmark) [16]. Pa14 was grown in 2YT [17].

### 2.2. Sequence Analysis

The sequence alignment obtained with ClustalW [18] was manually edited and visualized with Jalview [19]. The amyloïdogenic regions, i.e., the regions with tendency to self-assemble into a cross β–structure that forms the core of amyloid fibers, were predicted with the amylpred2 method [20]. Hydrophobicity profiles were obtained at the ExPASy server using the Eisenberg scale with default parameters [21].

### 2.3. Gene Expression Quantification

RNA-Seq data obtained previously to compare the transcriptomes of *A. fumigatus* growing under biofilm and liquid planktonic conditions [22], sporulating cultures [23], and in vivo data obtained here with mice were used to quantify the expression of the different hydrophobins as described previously [23]. For each dataset raw Illumina reads were quality and adapter trimmed using Trim Galore [24] with a quality cut-off of 20 and a minimum length cut-off of 30 bp. Quality trimmed reads were then mapped against the reference *A. fumigatus* Af293 genome using TopHat2 with the default setting [25]. Read counts and RPKM (Reads Per Kilo bases per Million reads) expression values for each gene were calculated using GFOLD [26].

For in vivo RNA-Seq data, eight-week-old OF1 male mice weighing approximately 28 g (Charles River Laboratory, L’Arbresle, France) were used. Mice were immunosuppressed with cyclophosphamide monohydrate (Sigma, St. Louis, MO, USA) injected intraperitoneally on days −3 and −1 (200 mg per kg of mouse) and cortisone acetate (Sigma) injected subcutaneously on days −3 and −1 (112 mg cortisone acetate per kg of mouse). Before conidial inhalation (day 0), mice were anesthetized with an intramuscular injection of 0.2 mL of a solution containing 10 mg/mL ketamin (Imalgene^®^ 1000, Merial, France), 1 mg/mL xylazin (Rompun^®^, Bayer Health-Care, Leverkusen, Germany) per mouse. Each mouse was inoculated intranasally with 6 × 10^7^ conidia (30 µL per mice of Tw-H_2_O conidial suspension at 2 × 10^9^ conidia per mL). On day 3, mice were euthanized by CO_2_ and lungs were removed and frozen in liquid nitrogen. After grinding the lungs in a mortar with liquid nitrogen, RNA was extracted with the mirVanaTM miRNA Isolation Kit (Ambion^®^ by Life Technologies, Hong Kong, China), quantified on a Nanodrop instrument (Thermofisher, Waltham, MA, USA), and quality controlled in an Agilent Bioanalyser 2100 (Agilent, Santa Clara, CA, USA).

### 2.4. Mutant Constructions

The ORF sequences of each *ROD* were replaced by the hygromycin or chlorimuron β-recombinase resistance cassette (*HPH*^R^ or Chlori^R^ β-rec) [27,28] through double crossing-over of the upstream and downstream borders, producing the Δ*rod* strains (Table S1; Figure S1A–G). Transformation of parental strain with the DNA construct by electroporation was performed as previously described [29]. The resulting transformants were analyzed by diagnostic PCR and Southern blot using the DIG probe protocol (Roche Diagnostics, Hong Kong, China). To construct multiple deletion strains, a previous-deletion strain was cultivated in the presence of 2% xylose-containing minimal medium that allows the excision of the selection marker, by recombination of the *SIX* recognition regions. A proper excision of the selection marker in the excised strain was then confirmed by PCR blot analysis before transformation of the mutant with a new replacement cassette.

### 2.5. Localization of Hydrophobins

#### 2.5.1. RodC-Flag Construction

The parental *RODC* ORF was replaced by a *RODC* ORF containing a Flag-tag inserted between the DNA bases T264 and G265, corresponding to the G88 and G89 amino acids in the C3–C4 loop of RodC. This loop sequence is very similar to the C3–C4 loop of RodA, known to be disordered as assessed by NMR [30]. The Flag-tag DNA sequence added was AGC GGA GAC TAT AAG GAC GAT GAC GAT AAG AGC GGA, codifying for the Flag-tag protein sequence SGDYKDDDDKSG [31].

The DNA construct to replace RodC for RodC-Flag was joined with four DNA fragments, called a, b, c, and d, are described in Figure S1 (HA). Fragments a, b, and d were amplified by PCR using the following pairs of primer numbers: 9-29, 30-31, and 32-12, respectively (Table S1). Fragment c was digested by FspI from plasmid pSK485 followed by DNA band gel purified [23]. The four fragments were assembled to each other with the GeneArt Seamless cloning and assembly kit (ThermoFisher). Transformation of parental strain with the DNA construct by electroporation was performed as previously described [29]. Southern blot was performed to check the correct insertion of the RodC-Flag in the transformants (Figure S1(HB,C)).

Conidia of RodC-Flag were grown on MM + 2% xylose to remove de ChloriR β-rec cassette followed by gDNA extraction and PCR with primers 33 and 34 for DNA sequencing.

#### 2.5.2. Recombinant RodA, RodB, and RodF

Recombinant RodA (rRodA) expression and purification has been described [30]. Like rRodA, recombinant RodB (rRodB), and RodF (rRodF) were produced as fusion proteins with N-terminal hexa-histidine tagged ubiquitin (h6Ubi) in *Escherichia coli* (BL21 strain). The sequence contains a deubiquitinase UBP40 cleavage between the h6Ubi and the Rod proteins.

The rRodB plasmid, which is based on the pHUE vector [32] was a kind gift of M. Sunde (University of Sydney). The rRodF plasmid based on a pET-28b(+) vector was purchased from Proteogenix (Schiltigheim, France).

Protein expression, purification in denaturing conditions by nickel affinity chromatography, in vitro oxidative refolding, cleavage with the deubiquitinase UBP40, and further purification by nickel affinity and reverse-phase chromatographies were performed following the published protocol used for rRodA [30].

The sequences of recombinant hydrophobins corresponded to the full-length proteins without the corresponding predicted N-terminal secretion peptide, namely residues 17–140 for RodB (AFUA_1G175250) and 19–212 for RodF (AFUA_5G03280). Both sequences contained an extra serine N-terminal residue that arose from cloning.

#### 2.5.3. Production of Polyclonal Antisera against RodA, RodB, and RodF

Polyclonal antisera against RodA and RodB had been produced in mice by Paris et al. [6]. However, they recognized the two hydrophobins only on Western blots, not by immunofluorescence. For this reason, we produced new polyclonal antisera against recombinant hydrophobins without their signal peptide.

rRodA [30], rRodB, and rRodF, without the signal peptide, were used to immunize BALB/cByJ mice intracutaneously. Several booster injections (10 µg per mouse) were performed every two weeks in the presence of complete (first injection) or incomplete Freund’s adjuvant. Immunization was followed by a direct enzyme-linked immunosorbent assay (ELISA) method with peroxidase-conjugated anti-mouse IgG. Mice were sacrificed after the ELISA test gave positive results at 1:2500 serum dilutions using 1 µg recombinant proteins as antigens, as previously described (Table S2) [33].

### 2.6. Conidia Permeabilization

Conidia were permeabilized as described previously [34]. Briefly, pFA-fixed conidia were permeabilized by successive incubations in Glucanex (Novozym, Bagsværd, Denmark) for cell wall degradation, in the detergent Nonidet P-40 (Sigma) and, finally, in methanol.

### 2.7. FITC Labeling, Immunofluorescence, and Immunoblotting

Permeability of the conidia to FITC was investigated by incubating 200 µL of an aqueous suspension of 10^6^ conidia with 30 µL of FITC solution (0.1 mg/mL in Na_2_CO_3_ 0.1 M pH 9) for 3 h at room temperature in darkness. The conidia were washed three times with Tw-H_2_O before observation under fluorescent light at 518 nm.

Immunofluorescence was done as described in Beauvais et al. [35]. Briefly, conidia or mycelium were fixed with p-formaldehyde 2.5% (pFA) overnight at 4 °C and washed with phosphate-buffered saline pH 7 (PBS) containing 0.1 M NH_4_Cl and then with PBS. For Flag detection on RodC-Flag, samples were immunolabeled with anti-Flag M2 monoclonal antibodies (Mab) (20 µg/mL; Sigma F3165), followed by incubation with anti-mouse IgG conjugated to Alexa488 (anti-mouse IgG-A488, 1:500 dilution; Sigma). For RodA, RodB, and RodF detection, samples were immunolabeled respectively by their corresponding antisera (1:250–1:500 dilutions) and the label was revealed with the anti-mouse IgG-A488.

For immunodetection on Western blotting, conidia, and mycelium were disrupted using 0.17 mm (for conidia) and 1 mm (for mycelium) beads for two minutes at 4 °C using a Fast-Prep cell breaker (MP Biomedical). Cell walls were obtained by centrifugation at 4000× *g* and membranes at 13,000× *g*. Class I hydrophobins from lyophilized conidia, cell wall or membrane fractions, were extracted by pure formic acid for 10 min to 2 h at 4 °C or by trifluoroacetic acid for 10 min at lab temperature [6]. After centrifugation at 13,000× *g*, the supernatants were evaporated under nitrogen and the resulting materials were washed with water by evaporation under nitrogen. The corresponding 13,000× *g* pellets were extracted by reducing and denaturing SDS-mercaptoethanol buffer (Tris-HCl 62 mM pH 6.8 containing 2% SDS and 5% β-mercaptoethanol). All fractions were analyzed by SDS-PAGE on a 15% polyacrylamide gel, under reducing conditions (LaemmLi, BioRad Mini-Protean Tetra Cell instruction manual). Proteins were transferred by Western blotting. RodC-Flag was detected by anti-Flag M2 Mab (10 µg/mL), and RodA, B and F by their respective anti-serum (1:1000 dilution), followed by incubation with anti-mouse IgG conjugated to peroxidase (1:2000 dilution; Sigma). For detection, the ECL chemiluminescence method of Amersham (GE Healthcare Life Sciences, Velizy-Villacoublay, France) was used.

### 2.8. Conidiation Measurement and Survival

Conidiation quantification and survival were conducted as previously described [36]. Briefly, conidia were recovered from 10 mL of malt agar slants with 5 mL Tw-H_2_O and quantified. To investigate conidial survival, conidia were kept dry or in Tw-H_2_O for up to two months at 37 °C. Germination was quantified on malt-agar.

### 2.9. Hydrophobicity Measurements

One milliliter of H_2_O was added to a three-weeks old malt tube culture, the surface of the tube was gently scraped with an inoculation loop, vortexed for 30 s, and the water containing conidia was carefully recovered using a Pasteur pipet. One milliliter of Tw-H_2_O was then added to the tube, which was vortexed for 30 s and Tw-H_2_O containing conidia was recovered. The percentage of hydrophobic conidia was estimated from the ratio of conidia counted in Tw-H_2_O solution vs. the total number of conidia.

### 2.10. Analysis of the Conidial Surface by Atomic Force Microscopy (AFM)

For AFM experiments, conidia were immobilized by mechanical trapping into isoporous polycarbonate membranes of 3 µm pore size (Millipore, Burlington, MA, USA), close to the dimension of the conidia. After filtering a spore suspension (20 mL; 10^6^ cells per mL), the filter was carefully rinsed three times in deionized water and cut (1 cm × 1 cm). The lower part was carefully dried on a sheet of tissue and the specimen was attached to a steel sample puck using a small piece of adhesive tape. A droplet of liquid was rapidly added on the filter to avoid cell desiccation and the mounted sample was then transferred into the AFM liquid cell. Experiments were performed in contact mode in liquid and at room temperature using a Nanoscope 8 Multimode AFM (Bruker, Santa Barbara, CA, USA). Oxide-sharpened microfabricated silicon nitride (Si_3_N_4_) AFM probes with triangular cantilevers of stiffness 0.01 N/m were selected (MSCT, Bruker, Santa Barbara, CA, USA).

### 2.11. Adherence Assays

Conidia were washed twice in Tw-H_2_O and suspended in H_2_O. The ability of conidia to adhere to polystyrene was tested by incubating 300 µL conidia (10^6^/mL) for 1 h in 48-well polystyrene plates (TPP, ThermoFisher). The plates were then washed several times with water and the remaining adherent conidia were quantified by adding 300 µL of GYE medium supplemented with the redox indicator resazurin (Sigma) (GYE-resa) to assess the biomass growth as previously described [37]. Conidia (15 µL) from the starting and from the remaining non-adherent solutions were incubated at 37 °C in 300 µL GYE-resa in 48 TPP wells. Growth was estimated by measuring OD (Optic Density) at 600 nm [37].

### 2.12. Resistance of Conidia to Glass Beads Disruption

*A. fumigatus* conidia (10^7^/mL) in 0.5 mL Tw-H_2_O were mixed with 0.5 mL (packed volume) of 0.17 mm glass beads. Conidia were then disrupted for 1 min in a Fast-prep cell breaker (MP Biomedical, Santa Ana, CA, USA). 5 × 10^3^ conidia/mL were plated (100 µL) on GYE plates in triplicate. The plates were incubated at 37 °C 24 to 36 h, colonies were counted, and survival rates were calculated by comparison with the non-disrupted plated conidial suspension.

### 2.13. Drug Susceptibility Testing

Minimal effective concentration (MEC) of caspofungin, a cell wall β-1,3-glucan synthesis inhibitor, and minimal inhibitory concentration (MIC) of posaconazole, SDS and H_2_O_2_ for the susceptibility to detergent and oxidative damages, were determined in MM-reza according to the Clinical Laboratory Standards Institute M38-A2 protocol (NCCM) by the microdilution method in 96-well plates [37].

To test the susceptibility to Congo red (CR) and calcofluor white (CFW), 500 conidia were spotted on six-well microplates (tissue culture plates, Sigma Aldrich) containing serial dilutions of CR or CWF in MM agar medium and incubated at 37 °C [36].

### 2.14. Aerial Static Biofilm and Shaken Submerged Conditions

Mycelia under aerial static biofilm or planktonic conditions were obtained as previously described [14]. Briefly, 10^6^/mL conidia were inoculated in 20 mL liquid, or agar GYE covered by cellophane (DryEase cellophane, Invitrogen, Carlsbad, CA, USA) at 30 °C for 24 h and the mycelium dry weight was quantified. The hydrophobicity of the biofilm was tested by placing 10 µL drops of 0.2% SDS in 50 mM EDTA on the surface of the colony [38].

For immunofluorescence, biofilms were pre-formed in liquid GYE eight-well glass bottom Ibidi μ-slides (10^6^/mL conidia, 250 µL, 37 °C 18 h).

The ability of the bacteria *P. aeruginosa* Pa14 to adhere on the mycelium of hydrophobin mutants and the parental strain ku80 was evaluated by mixing in PBS bacteria (2 × 10^7^/mL) and hyphae grown in liquid GYE, and observing the binding on a fluorescence microscope (GFP filter, exc. 470 nm, em. 509 nm).

### 2.15. Generation and Culture of Human Dendritic Cells

Human DCs were generated from circulating monocytes as previously described [15]. DCs were cultured with pFA-fixed conidia at a 1:1 ratio for 48 h. For flow-cytometry analysis of DCs, FITC-conjugated MAb to CD86 and APC-conjugated MAb CD83 (all from BD Biosciences, San Jose, CA, USA) were used.

### 2.16. Virulence Assays in Mice

For virulence assays, four-week-old OF1 female mice (each 18–20 g, Charles River Laboratory, L’Arbresle, France) were immunosuppressed with Kenacort^®^ Retard (Triamcinolone acetonide; Bristol-Myers Squibb), injected subcutaneously on day −1 (40 mg Kenacort per kg of mouse). Before conidial inhalation (day 0), mice were anesthetized with an intraperitoneal injection of 0.2 mL of a solution containing 10 mg/mL ketamin (Imalgène^®^ 1000, Merial, Lyon, France) and 1 mg/mL xylazin (Rompun^®^, Bayer Health-Care, Germany). Mice were inoculated intranasally with a suspension of 2 × 10^6^ conidia in 20 µL PBS-Tw-H_2_O per mouse. Non-infected control immunosuppressed mice only received 20 µL of PBS-Tw-H_2_O. A daily monitoring of the body weight of the mice was conducted and a loss greater than 25% of the initial body weight was considered as a limit of pain not to be exceeded. Any animal that passed this threshold was immediately euthanized.

### 2.17. Statistical Analysis

Data are reported as means ± s.e.m. Comparisons were performed with Graph Pad Prism 3.0 (San Diego, California, USA) or JMP (University of Georgia, Athens, Georgia, USA) softwares and analysis of variance statistical tests.

## 3. Results

### 3.1. Sequence Analysis

Three major characteristics define hydrophobins: the presence of the hydrophobin cysteine motif, the hydrophobicity profile, and the capacity (or not) to form amyloid fibers that divide hydrophobins into different classes. Seven putative hydrophobin genes, named *RODA* to *RODG*, were identified in the *A. fumigatus* genome. The proteins encoded contained 125 to 211 amino acids. Except RodD and RodE, all the proteins contained an N-terminal signal peptide.

**Figure 1:**
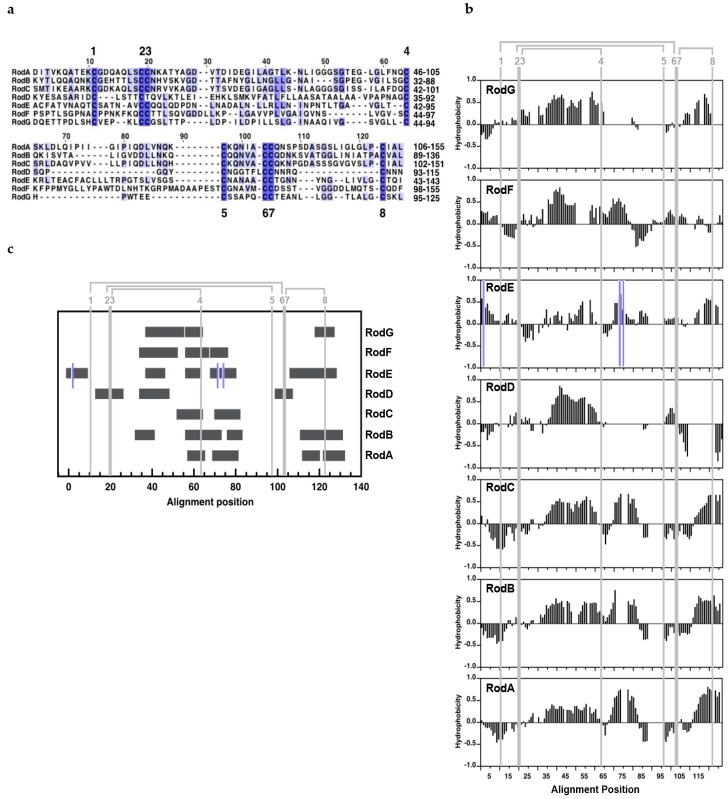
Alignment of *A. fumigatus* hydrophobins: (**a**) sequence; (**b**) hydrophobicity profiles; (**c**): amyloidogenicity predicted with amylpred2 (Tsolis *et al.*, 2013). Alignments include only the regions with the hydrophobin idiosyncratic Cys-residues motif. The positions of hydrophobin Cys-residues are labeled in their order of appearance from 1 to 8 throughout this work. The disulfide topology is shown in grey. In (**a**), the alignment position is shown on top and sequence numbering on the right; the intensity of the blue color reflects the degree of identity. In (**b**,**c**), the positions of the three extra Cys-residues in RodE are marked with a blue line.

RodA, RodB, and RodC were close homologues with over 40% sequence identities and 50% similarities between them (Figure 1a; Table S3). The solution NMR analysis of the secondary structure of RodA, of its disulfide topology [30] and the high level of sequence similarity indicates that RodB and RodC monomers, like RodA monomers, contain a barrel organized around the S-S bridges and the typical hydrophobin S-S topology (C1–C6, C2–C5, C3–C4, C7–C8, [39]). Although these three hydrophobins are predicted to have a C-terminal GPI-anchor that could be at the origin of their linkage to the cell wall, two lines of evidence indicate that the proteins are actually not GPI-anchored: the predicted cleavage site, indeed, is located between Cys-residues C7 and C8, which would disrupt a conserved disulfide bridge that is important to stabilize the structure of the proteins; moreover, it has been shown that the C-terminus of RodA extracted from conidia corresponds to that of the full-length protein [30].

RodD-G show very low similarity between them and with RodA-C (Figure 1a; Table S3). RodD has several unusual features that render its classification as a hydrophobin controversial. This protein does not have a secretion signal, has an extremely short C3–C4 loop and a very long C-terminal tail (132 residues) after cysteine C8, has an atypical hydrophobicity profile showing a highly hydrophilic C7–C8 region in contrast with class I and class II hydrophobins (Figure 1b) and, most importantly, it lacks one cysteine (Cys3 or Cys4, based on the position) of the first of the two CC doublets that define hydrophobins. The absence of a disulfide bridge between cysteine residues C3–C4 would completely disrupt the characteristic β-barrel observed in class I and class II hydrophobins [39,40,41,42], which has a high curvature constrained by the S-S bonds.

RodE has three extra-cysteines, two of them within the characteristic hydrophobin Cys pattern, and no signal peptide, and was previously excluded from the hydrophobin family [2]. However, its hydrophobicity profile is conserved with class I hydrophobins represented by RodA, RodB, and RodC (Figure 1b). For this reason, and because the hydrophobin fold could, in principle, accommodate these extra-cysteines, we designated this protein as a presumed class I hydrophobin.

RodF and RodG qualify as hydrophobins because both proteins contain a signal peptide and the conserved Cys-pattern. Although RodF contains one extra cysteine residue, it is located in the N-terminal secretion peptide. Its hydrophobicity profile resembles that of class I hydrophobins except on the C7–C8 region, where class I hydrophobins show a highly hydrophobic sequence close to C8 (Figure 1b). Finally, RodG shows a class I hydrophobicity pattern, but an unusually short C5–C6 region for class I or class II hydrophobins and unusually long C3–C4 region for a class II hydrophobin.

Rodlets formed by class I hydrophobins show the hallmarks of amyloid fibers [1,43]. The amyloidogenicity that is the tendency of short peptide sequences (4–7 residues) to self-associate into β-sheets that constitute the cross β-structure at the core of amyloids can be predicted from the sequence. The seven hydrophobin sequences of *A. fumigatus* analyzed in this work show two or more amyloidogenic regions, indicating that it cannot be excluded as the proteins might associate into amyloids, like class I hydrophobins (Figure 1c).

### 3.2. Expression Analysis

**Figure 2:**
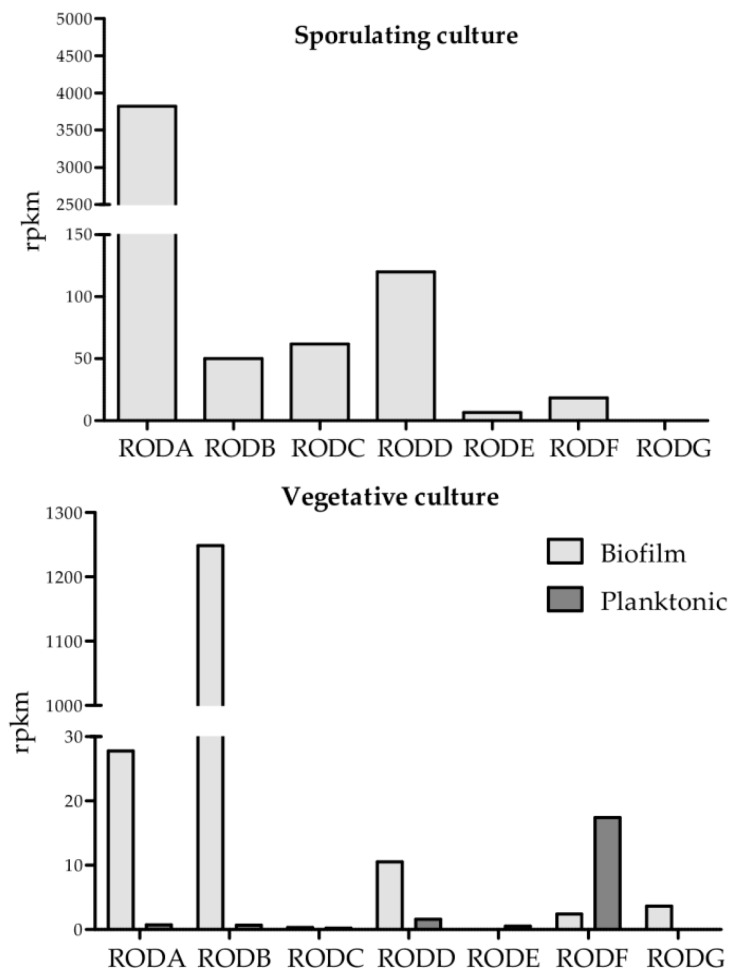

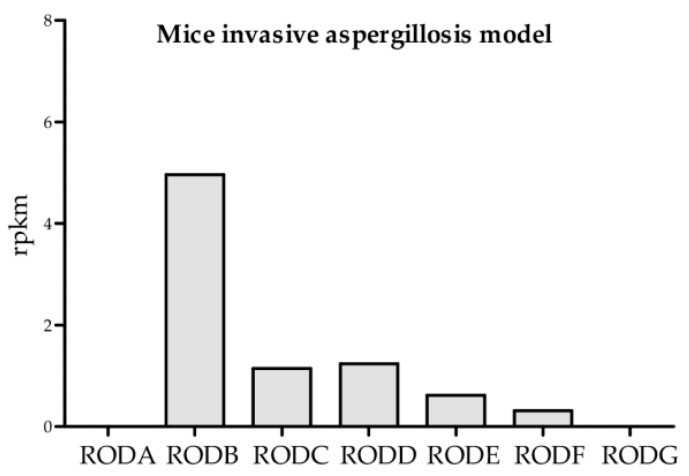
Expression of the different hydrophobin genes under different conditions.

Expression analysis of all hydrophobin genes showed that the most highly expressed genes were *RODA* and *RODB* (Figure 2). *RODA* was the most highly expressed hydrophobin in sporulating culture, whereas *RODB* was highly expressed in the biofilm condition and in vivo. In comparison, the other hydrophobin genes were poorly expressed at far lower levels. *RODC* was primarily expressed in sporulating culture and *RODF* was only expressed at moderate levels in planktonic and sporulating conditions. *RODE* and *RODG* were either not expressed or expressed at extremely low levels. *RODD* presented low expression in biofilm, but high expression in sporulating culture.

### 3.3. Localization of Hydrophobins

**Figure 3:**
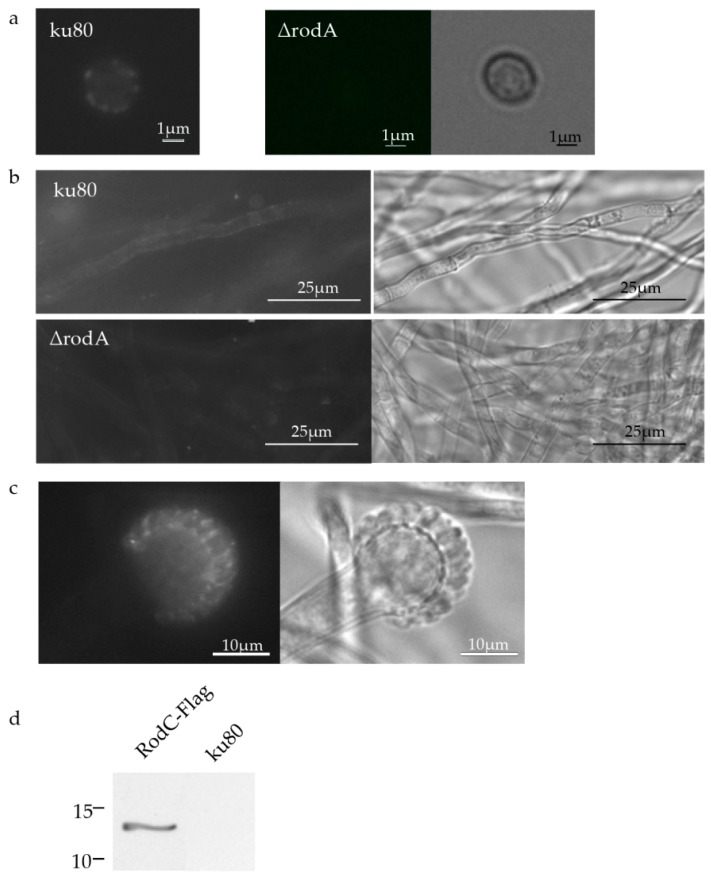
Localization of RodA and RodC. Immunofluorescence localization of RodA on conidia (**a**) and biofilm (**b**) cell walls; and on phialides (**c**) of ku80 (∆*rodA* was used as a negative control) using the anti-recombinant RodA polyclonal antiserum; Immunoblotting localization of formic acid-soluble material of RodC-Flag and ku80 (negative control) conidia using an anti-Flag monoclonal antibody (**d**).

Using a newly-prepared polyclonal mouse anti-RodA directed against recombinant RodA, it was shown by Western blot (as shown also by Paris et al. [6]) and by immunofluorescence that RodA was present both on the surface of the conidium (Figure S2, Figure 3a), in the biofilm (Figure 3b) and in the phialides of the *A. fumigatus* head (Figure 3c).

Western blots with the newly-prepared polyclonal antibody anti-RodB confirmed that RodB was present in the conidium cell wall (Figure S2; as shown also by Paris et al. [6]), but absent from hyphae of planktonic or biofilm cultures (data not shown). However, like the previous anti-RodB Abs, the new one did not allow the detection of RodB by immunofluorescence on conidia or mycelium, even after permeabilization.

The use of an anti-Flag Ab showed that RodC was also present in the conidial cell wall and can be extracted by formic acid like other class I hydrophobins (Figure 3d).

The localization of RodF was unsuccessful when using an anti-RodF polyclonal antibody. This result suggested that the amount of protein was too low in *A. fumigatus* to be detected by an antibody, which was positive at a 1:2500 dilution against the recombinant RodF (data not shown). Taking into account this last result, localization of RodE and RodG was not attempted because these two genes are too weakly expressed in the growth condition tested. Since RodD was not considered as a hydrophobin, its localization was not attempted in this study.

In conclusion, RodA, B, and C were present in the conidia, while RodA was the only one present in biofilm in spite of the RNA-Seq data showing a high expression of *RODB*.

### 3.4. Hydrophobin Mutant Analysis

Single mutants and multiple Δ*rodBC*, Δ*rodBCD*, Δ*rodBCDE*, Δ*rodBCDEF*, Δ*rodBCDEFG*, and Δ*rodBCDEFGA* were constructed as described in the Material and Methods section.

**Figure 4:**
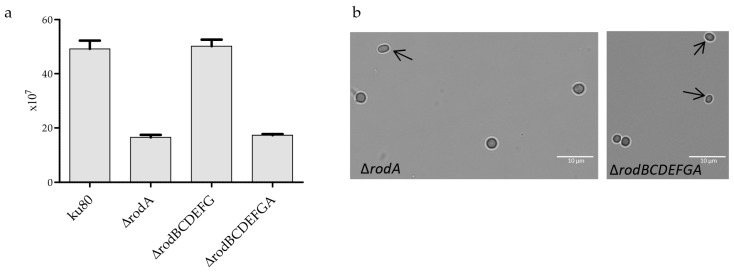
Conidiation of hydrophobin mutants. Sporulation of ku80 and hydrophobin mutants, quantification of the amount of conidia was conducted from three different malt agar slants (**a**); morphology of *RODA* deleted conidia, showing some conidia with an oval shape (arrow) (**b**).

Deletion of *RODA* led to a 65% decrease in sporulation and conidia formed cell clumps (Figure 4a, [10]). The presence of RodA on the surface of the phialides (Figure 3c) is required for proper conidiogenesis. *A. fumigatus c*onidia are normally round, but oval shapes were observed in Δ*rodA* and Δ*rodBCDEFGA* (Figure 4b).

**Figure 5:**
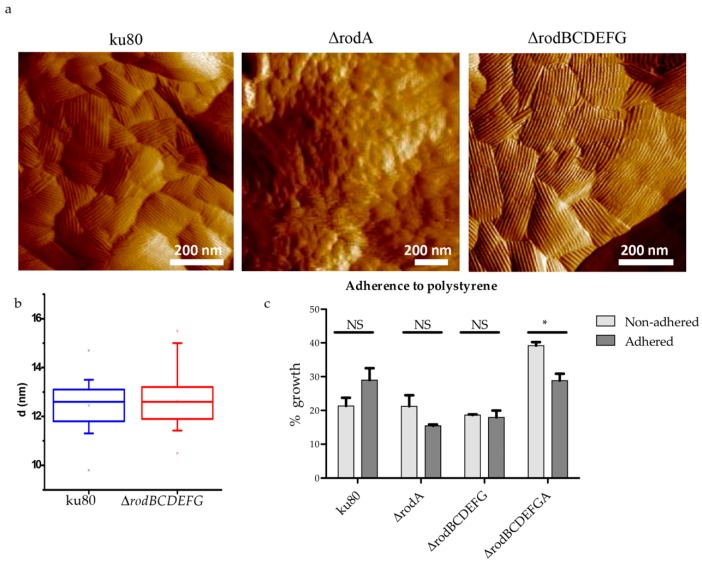
Structure of the conidial cell wall surface of ku80, ∆*rodA*, and ∆*rodBCDEFG* conidia. AFM images show the presence of rodlets on the surface of ku80 and ∆*rodBCDEFG*, whereas the surface of conidia deleted in *RODA* is amorphous (**a**); box plots showing the distance between two rodlets on ku80 and ∆*rodBCDEFG* conidia (*n* = 20 in both cases) (**b**); and adherence to polystyrene plates of hydrophobin mutant and parental strain conidia (**c**). NS: not significant, * *p* < 0.05.

The analysis of rodlet formation and conidia hydrophobicity performed with all the mutants showed that only RodA was responsible for both (Figure 5a,b; Figure S3). The low hydrophobicity of Δ*rodA* and Δ*rodBCDEFGA* slightly decreased the adherence of conidia to polystyrene plates, especially in the absence of the other hydrophobins (Δ*rodBCDEFGA*) (Figure 5c).

**Figure 6:**
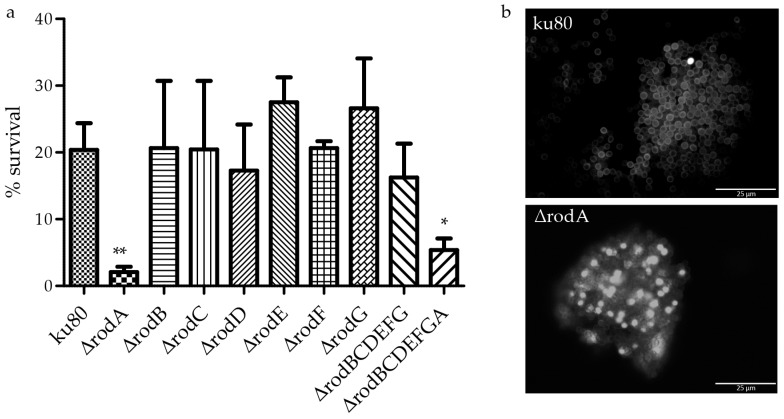
Resistance and permeability of the ∆*rodA* conidial cell wall. After physical disruption of conidia by 0.17 mm beads for 1 min, conidial survival was estimated by CFU, * *p* < 0.05 (**a**); FITC labeling of ∆*rodA* and ku80 conidia showing a modification of the cell wall permeability seen by the intracellular staining of many ∆*rodA* conidia (**b**).

Compared to WT, Δ*rodA* conidia were hypersensitive to physical insult, as evidenced by the lower survival of Δ*rodA* after physical disruption of conidia by beads (Figure 6a), suggesting that their cell wall was significantly weaker than that of the parental strain. Δ*rodA* conidia were intracellularly labeled by FITC, whereas the fluorochrome bound exclusively to the cell wall of the parental strain ku80. These data suggested an increased permeability of the cell wall of the mutant (Figure 6b). These results can be related to the different shapes of the *RODA* deleted conidia, showing an increase in the plasticity of the conidial cell wall (Figure 4b). The deletion of *RODA* in addition to the deletion of the other hydrophobins (Δ*rodBCDEFGA*) slightly decreased the survival in air (ku80 82% (±1.5), Δ*rodA* 89% (±2.5), Δ*rodBCDEFG* 88% (±2.3), and Δ*rodBCDEFGA* 73% (±1.9, *p* < 0.05). After two months in water, the survival of all hydrophobin mutant conidia was similar to ku80 (95%).

The softening and structural modifications of the cell wall of Δ*rodA* and Δ*rodBCDEFGA* suggested that these mutants could behave differently to the parental strain during germination or in the presence of antifungal drugs. We found that, relative to germination, all hydrophobin mutants showed the same behavior than ku80 (Figure S4). Similarly, all mutants behaved like the parental strain against posaconazole, caspofungin, SDS, and H_2_O_2_ (Table S4). In contrast, Δ*rodA* and Δ*rodBCDEFGA* were highly resistant to CR and CFW, indicating that the structural modifications brought by *RODA* deletion had an impact on the resistance of conidia to this category of inhibitors (Table 1).

We then analyzed the role of hydrophobins in aerial and static biofilm conditions. By RNA-seq, *RODB* was found highly expressed in biofilm conditions but, immunologically, RodB was not detected in mycelium. The analysis of the phenotype of Δ*rodB* showed that, in accordance with the immunolocalization, the growth of the mutant was like ku80 under biofilm and planktonic conditions. Increasing the number of hydrophobin gene deletion did not change the structure and biomass of the mycelium produced under biofilm or planktonic conditions, suggesting that hydrophobins were not essential for biofilm formation in *A. fumigatus* (Figure S5A). Moreover, hydrophobins did not play a role in biofilm hydrophobicity as observed by the similar aspect of the detergent drop on the surface of hydrophobin mutants and ku80 (Figure S5B). Indeed, if biofilms formed by the mutants were more hydrophilic, the droplets would soak into the colony [38]. The adhesion of *P. aeruginosa* on the mycelia of hydrophobin mutants and ku80 was not modified (Figure S5C), suggesting that the surface ionic characteristics did not change [17]. Consequently, hydrophobins did not play a role in the establishment of the biofilm.

**Figure 7:**
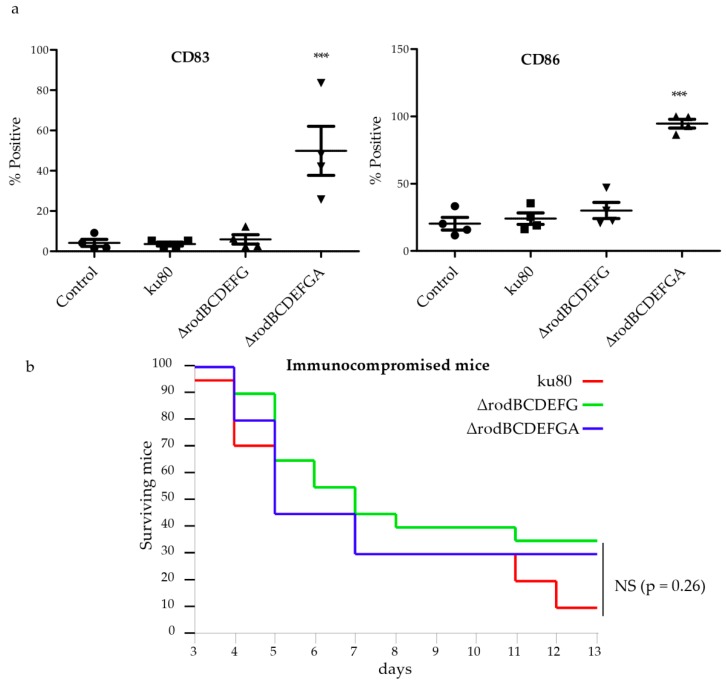
Virulence of hydrophobin mutant conidia. Effect on the maturation of human DCs (**a**); control represents non-stimulated DCs; infection of immunocompromised mice (**b**). *** *p* < 0.001; NS: non-significant.

During infection, RodA masked the recognition of immunogenic motifs of *A. fumigatus* conidia by host innate cells, such as DCs [7]. The capacity of other hydrophobins to play a similar role was tested. Conidia from ku80, Δ*rodBCDEFG*, and Δ*rodBCDEFGA* were used to study the maturation of DCs. Like Δ*rodA*, Δ*rodBCDEFGA* induced the maturation of DCs, as demonstrated by the significantly enhanced expression of CD83 and the co-stimulatory molecule CD86. In contrast, ku80 and Δ*rodBCDEFG* were immunologically inert (Figure 7a).

We tested the virulence of Δ*rodBCDEFG* and Δ*rodBCDEFGA* mutants in immunocompromised mice. The virulence of the two mutants was similar to the WT (Figure 7b).

## 4. Discussion

Seven hydrophobins were found in *A. fumigatus*, although classification of RodD is controversial. In the closely-related species *A. nidulans*, six hydrophobins were found in the genome [38], three of which (AnRodA, DewA, and DewB) belong to class I, as in *A. fumigatus*. However, the *A. nidulans* DewC hydropathic profile is very similar to AnRodA, and could also be assigned to class I. This is similar for RodE, which can also be assigned to class I in *A. fumigatus.* Only *A. nidulans* class I AnRodAp/DewBp and *A. fumigatus* class I RodAp/RodBp/RodCp show significant sequence identities (45–75%). As in the case of *A. fumigatus*, *A. nidulans* also has class III hydrophobins DewD and DewE. These hydrophobins show little identity between them and with the other *A. nidulans* or *A. fumigatus* class I hydrophobins (20–30%).

In *A. fumigatus* and *A. nidulans*, class I hydrophobin genes are mainly expressed in sporulating culture, which correlates with the development of phialides and conidia [38]. Moreover, the corresponding proteins were localized on the cell wall of conidia. Class I hydrophobin genes in *A. nidulans* and in *Beauveria bassiana* are not expressed in vegetative mycelium [38,44], whereas in *A. fumigatus*, *RODA* and *RODB* are also expressed in biofilm conditions, despite our immunolocalization studies showing RodA, but not RodB, in biofilm. All class III genes in both *Apergillus* sp., have low expression during vegetative growth. RodD, a cys-rich protein, did not present the typical hydropathy profile and cysteine pattern of hydrophobins. The gene is expressed in sporulating culture and biofilm, but 30 to 125 times less than *RODB*. In fungi, another class of proteins containing a CFEM domain of eight cysteines pattern have a signal peptide [45,46]. The other well-characterized six-eight cysteine-containing domains are the epidermal growth factor (EGF)-like domains. These proteins are involved in biogenesis and/or maintenance of the biofilm structure and integrity, adhesion signal transduction, extracellular sensors, or cell surface receptors. However, RodD do not have a signal peptide, nor CFEM or EGF domains, and none of the functions related to the CFEM or EGF-like proteins were observed for RodD in *A. fumigatus*. Its role remains undetermined.

In *Aspergillus* sp., RodA is the only hydrophobin responsible for rodlet formation on the surface of conidia, whereas in *B. bassiana*, Hyd1 and Hyd2 interact to constitute the conidial rodlet layer [38,44]. In *A. nidulans*, DewA and DewB were able to produce rodlets to some extent upon expression controlled with the AnRodA promoter [38]. This result suggests that, in normal expression conditions, the hydrophobins of *A. nidulans* do not substitute for each other to form rodlets on the conidial surface, similarly to RodB and RodC in *A. fumigatus* conidia. In *A. fumigatus*, only RodA is responsible for all tested phenotypes: hydrophobicity, CW integrity, and phialides modifications, which impact on conidial production, survival, sensitivity to external aggressions (desiccation, drugs, and physical damages) and immune-inertia. Similarly, disruption of Mgp1 results in the loss of the *Magnaporte grisea* rodlet layer on the surface of conidia that participates in appressorium formation and virulence. Mutants in this gene also displayed reduced conidiation and viability [42].This feature is distinct in *A. nidulans* or *B. Bassiana* [38,44]. In these molds, indeed, the class I hydrophobins DewA–E or Hyd2 contribute along with AnRodA or Hyd1, respectively, to the conidial hydrophobicity. Furthermore, although Δ*hyd1* conidia form aggregates similar to those in *A. fumigatus* Δ*rodA,* the absence of Hyd1p did not change the conidial surface adhesion on hydrophobic surfaces*.* Only Δ*hyd2*, like *A. fumigatus* Δ*rodA*, had little effect on adhesion on hydrophobic surfaces*.* Surprisingly, *RODA* deletion increased the resistance of *A. fumigatus* to CR and CFW, a phenomenon that was not observed for *B. bassiana* hydrophobins. Nonetheless, in the same line of our observation for *A. fumigatus*, heterologous expression of the class I hydrophobin SC3 secreted by aerial hyphae of *Schizophyllum commune*, on the surface of *Picchia pastoris*, also reduced the yeast susceptibility to Congo red [47]. Exposure of other cell wall components on the surface of *A. fumigatus* conidia following rodlet deletion, such as β-(1,3)-glucan, could modify the drug uptake [7].

In *A. fumigatus* mycelium, hydrophobins do not play any role on the development or adherence of biofilm and its hydrophobicity, nor do these proteins modify the hyphal surface properties. Moreover, adherence properties of *P. aeruginosa* on ku80 and the null-hydrophobin mutant Δ*rodBCDEFGA* were similar. *RODB* was highly expressed during hyphal growth. However, high gene expression does not mean high protein production. This is different with SC3, which self-assembles at the outer wall surface and confers hydrophobicity to the aerial hyphae of *S. commune* [48]. Disruption of *SC3* modified the cell wall composition, which contains more mucilage (water soluble glucan) and less alkali-insoluble glucan, and led to a strong reduction of aerial hyphae formation and hydrophobicity.

Our results show that *A. fumigatus* RodA is the most important hydrophobin of the family. It was currently observed in *A. fumigatus* gene families that one member plays the major role in the cell and cannot be substituted by other members. For example, in the glucanosyl transferase GEL family, *GEL4* deletion is lethal and cannot be compensated for by other *GELS* [49,50]. Similarly, in the chitin synthase CHS family, phenotypic defects mainly result from the *CSMA* deletion [51]. In all fungi having α-(1,3)-glucan in their cell wall, mainly one α-(1,3)-glucan synthase over three or more proteins in the family is responsible for the synthesis of this polysaccharide and is only partially compensated by the others when deleted [52,53,54].

*RODB* was highly expressed in vivo. However, there was no difference of the dendritic cell’s response between Δ*rodBCDEFG* and parental strain, and there was no difference between Δ*rodBCDEFGA* and Δ*rodA* mutants. This lack of difference in the immune response has been many times associated to an absence of virulence difference in strains. It was demonstrated previously that ex vivo Δ*rodB* was killed by macrophages similarly to ku80, whereas Δ*rodA* showed a higher resistance [6]. However, previous results on Δ*rodA* [6,10], and our data on Δ*rodBCDEFGA* and Δ*rodBCDEFG*, showed that the virulence of hydrophobin mutants in our mice invasive aspergillosis model was similar to the parental strain. Similarly, infected rats with low doses of immunosuppressive drugs presented the same mortality when they were infected by WT or Δ*rodA* conidia [55]. However, in nature, Δ*rodA* and Δ*rodBCDEFGA* would never be dispersed because of their hydrophilic properties and, consequently, would never reach the respiratory tract and invade hosts.

In conclusion, RodA is the only essential hydrophobin in *A. fumigatus*, conditioning the structure, permeability, hydrophobicity, and immune-inertia of the cell wall surface in conidia. Moreover, deletion of *RODA* modifies the properties of the conidial cell wall surface and impacts on the drug sensitivity of the fungi. Herein we could not detect any role of *A. fumigatus* hydrophobins in biofilm formation and its hydrophobicity.

## Figures and Tables

**Table 1:** CMI values of hydrophobin mutants and the parental strain ku80 incubated in presence of congo red or calcofluor white for 48 h at 37 °C in MM medium. No statistically significant difference was found in the CMIs for each drug.

Strains	ku80	Δ*rodA*	Δ*rodBCDEFG*	Δ*rodBCDEFGA*
**MIC CR (µg/mL)**	100	>300	100	>300
**MIC CFW (µg/mL)**	80	150	80	150

## Supplementary Materials

The following are available online at www.mdpi.com/2309-608X/4/1/2/s1.
