# Supplementary material for: Role of Hydrophobins in Aspergillus fumigatus
Source: J Fungi (Basel). 2017 Dec 24;4(1):2. doi: 10.3390/jof4010002 (PMC5872305; doi:10.3390/jof4010002)
Supplement: Supplementary file 1 [file jof-04-00002-s001.pdf]

Table S1: Primers used in this study

**Name**	**Primer sequence**	**N°**
pUC19 NruI upstream RodA F	5′CCAGTGAATTCGAGCTCGGTAC**TCGCGA**GACGATGACTTGTAATATAT3′	1
Upstream RodA Chlo^{R}-β Rec R	5′GCATAATATGGTCCATCTAG**TGC**TGTGACGGTGGGGCTTTAAAAAGGGAG3′	2
Chlo^{R}-β Rec downstream RodA F	5′TATAGGACCTGAGTGA**TGC**GCTACTCGTCGCTTCTGCT3′	3
Downstream RodA NruI pUC19 R	5′CGCCAAGCTTGCATGCC**TCGCGA**GGGGGTCTACGATCTTTTCTGTGGATA3′	4
pUC19 FspI upstream RodB F	5′TGAATTCGAGCTCGGTAC**TGCGCA**CCCCCTTACCCTTTGGCTGGAGT3′	5
Upstream RodB HPH^{R}-βRec R	5′GACCTATAGGACCTGAGTGA**TGC**TGAGAATGGGTTAGAGGAGTTTGC3′	6
HPH^{R}-βRec downstream RodB F	5′TGGTCCATCTAG**TGC**GGACTTGAGGAGCGTTAATAATCGAGA3′	7
Downstream RodB pUC19 R	5′TACGCCAAGCTTGCATGCC**TGCGCA**AAGCCAGCGAGACCTACCGGGTT3′	8
puC18 SmaI upstream RodC F	5′GCCAAGCTTGCATGCC**CCCGGG**GACTTATTAATGAGGATTCTTTATGCTCTT3′	9
Upstream RodC HPH^{R}-βRec R	5′GGACCTGAGTGA**TGC**TATGACCAGTAATTATATTCTATGCCAGTC3′	10
HPH^{R}-βRec downstream RodC F	5′TGGTCCATCTAG**TGC**CTGGTTGTCACCTATCTATAATCATCTCTC3′	11
Downstream RodC SmaI pUC18 R	5′AATTCGAGCTCGGTAC**CCCGGG**AAGAGTATTACCTACATCTGTACGCGA3′	12
pUC18 upstream FspI RodD F	5′GCCAAGCTTGCATGCC**TGCGCA**TATATAGATGCATAGAGGTGATACCCA3′	13
Upstream RodD HPH^{R}-βRec R	5′GGACCTGAGTGA**TGC**GATATGATAAGACCGAGACAGATAAGATAA3′	14
HPH^{R}-βRec downstream RodD F	5′TGGTCCATCTAG**TGC**GGTTTCTTATAATCAATGGGCAAAGTA3′	15
Downstream RodD FspI pUC18 R	5′AATTCGAGCTCGGTAC**TGCGCA**GTAGAGTTTGTGTCTCTTTGGGATAGTAAC3′	16
puC18 FspI upstream RodE F	5′ GCCAAGCTTGCATGCC**TGCGCA**ATTTCAGTTGACTGTCTCCATCTTTAT3′	17
Upstream RodE HPH^{R}-βrec R	5′GGACCTGAGTGA**TGC**AGTTTGAGTCTGTGATTGTGATTGAGT3′	18
HPH^{R}-βrec downstream RodE F	5′TGGTCCATCTAG**TGC**ATACTTACTCGATATTAGAACGGACTCATT3′	19
Downstream RodE FspI pUC18 R	5′AATTCGAGCTCGGTAC**TGCGCA**AGTATAGGACTATAGGTAAGGTGTCCTCTG3′	20
pUC19 FspI upstream RodF F	5′AATTCGAGCTCGGTAC**TGCGCA**CATCATCTTCATTTCTTGTC3′	21
Upstream RodF HPH^{R}-βrec R	5′ATAGGACCTGAGTGA**TGC**TGTGTCGAAGTCGTAGTTGGCG3′	22
HPH^{R}-βrec downstream RodF F	5′TAATATGGTCCATCTAG**TGC**ATTCACTGGTCGGGGTGTCT3′	23
Downstream RodF FspI pUC19 R	5′GCCAAGCTTGCATGCC**TGCGCA**GGTGAAATATCTACTGTACA3′	24
pUC19 FspI upstream RodG F	5′AATTCGAGCTCGGTAC**TGCGCA**TAAGTGTCTATCATCCCACC3′	25
Upstream RodG HPH^{R}-βrec R	5′TATAGGACCTGAGTGA**TGC**TGTGGAGATAGAGGATGTTTGAGA3′	26
HPH^{R}-βrec downstream RodG F	5′ATAATATGGTCCATCTAG**TGC**GTACGCGGTTTTGAGGTTCAA3′	27
Downstream RodG FspI pUC19 R	5′GCCAAGCTTGCATGCC**TGCGCA**CCGCATCTCGTCTCTCGTTGCT3′	28
ORF RodC + flag tag R	5′TCCGCTCTTATCGTCATCGTCCTTATAGTCTCCGCTACCCCCAGCGAGGTTGCTCAGAAG3′	29
ORF RodC + flag tag F	5′GGTAGCGGAGACTATAAGGACGATGACGATAAGAGCGGAGGTTCCGGAATAAGCAGCAT3′	30
ORF RodC Stop Chlo^{R}-β Rec R	5′ GCATAATATGGTCCATCTAG**TGC**TTACGCAACGGATCCCAAAGCAATG3′	31
Chlo^{R}-β Rec Downstream RodC F	5′CCTATAGGACCTGAGTGA**TGC**GGTTATGATGGCATATGAACG3′	32
Upstream RodC screen F	5′ACTGGGTGGGAAAGATTGTG3′	33
Downstream RodC screen R	5′AGACAACGAAAAACGCGACT3′	34

Not underlined = pUC18, pUC19, HPH^{R}-βrec or Chlo^{R}-β Rec
Underlined = Hydrophobins borders or ORF DNA sequences
Bold = Partial or total restriction enzyme sequences
Double underlined = Flag tag sequence

Table S2: ELISA data (OD 492 nm) of new polyclonal antisera against recombinant hydrophobins rRodA, rRodB and rRodF without their signal peptide.

	**11:500 dilution**	**1:2500 dilution**
**Antisera anti-RodA**	2,778	2,127
**Antisera anti-RodB**	2,157	2,168
**Antisera anti-RodF**	2,281	1,774

Table S3: Percentage of identities (top) and similarities (bottom) between *A. fumigatus* hydrophobins.

	RodB	RodC	RodD	RodE	RodF	RodG
**RodA**	44.8<br>58.1	60.9<br>72.7	14.8<br>21.0	19.6<br>26.5	12.7<br>20.9	20.7<br>26.8
**RodB**		40.0<br>51.4	16.0<br>25.9	20.6<br>31.4	19.0<br>25.7	20.7<br>28.0
**RodC**			14.8<br>22.2	19.6<br>26.5	11.8<br>17.3	17.1<br>23.2
**RodD**				16.0<br>19.8	13.6<br>18.5	13.6<br>16.0
**RodE**					17.6<br>25.5	30.5<br>37.8
**RodF**						25.6<br>35.4

Table S4: CMI values of hydrophobin mutants and the parental strain ku80 incubated in presence of congo red or calcofluor white for 48 hr at 37°C in MM medium. No statistically significant difference in the CMIs for each drug.

Strains	ku80	Δ*rodA*	Δ*rodBCDEFG*	Δ*rodBCDEFGA*
**MIC posaconazole (µg/ml)**	0,25	0,25-0,5	0,25	0,25
**MEC caspofungin (µg/ml)**	0,25	0,25	0,25	0,25
**MIC H_2O_2 (mM)**	1-2	2	1-2	2
**MIC SDS (%)**	0,05	0,05	0,05	0,025-0,05

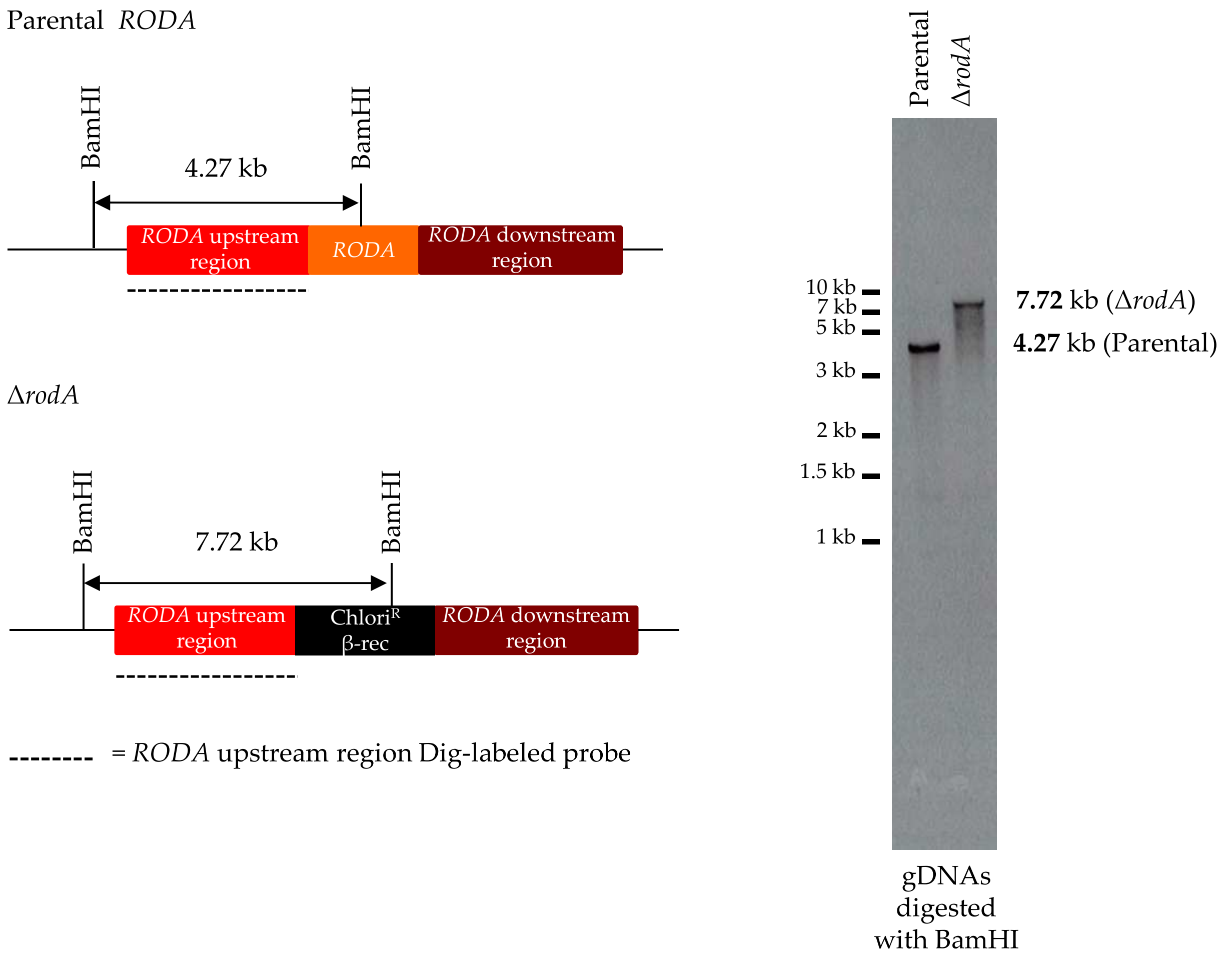

Figure S1A: Southern blot of the *A. fumigatus* Δ*rodA* strain

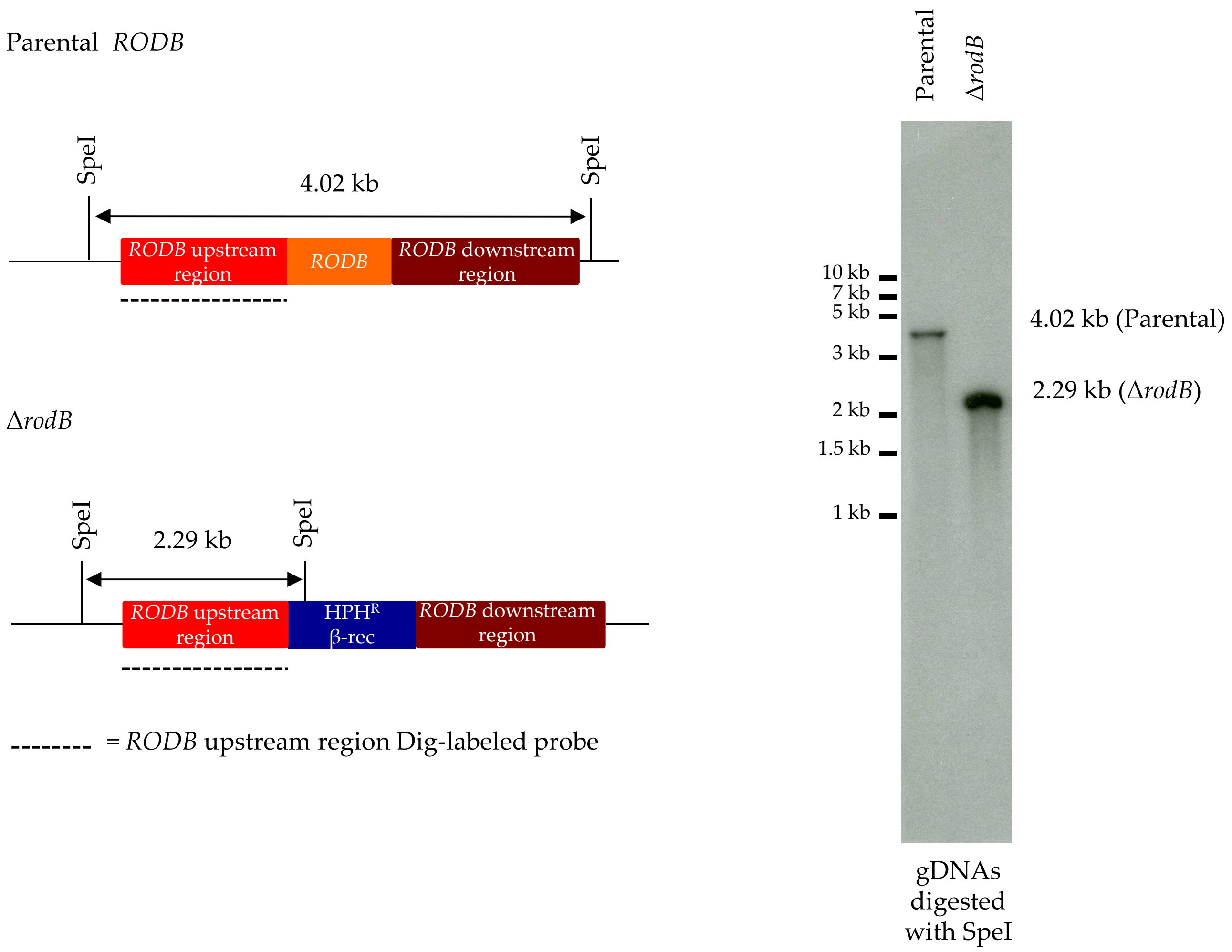

Figure S1B: Southern blot of the Southern blot of the *A. fumigatus* Δ*rodB* strain.

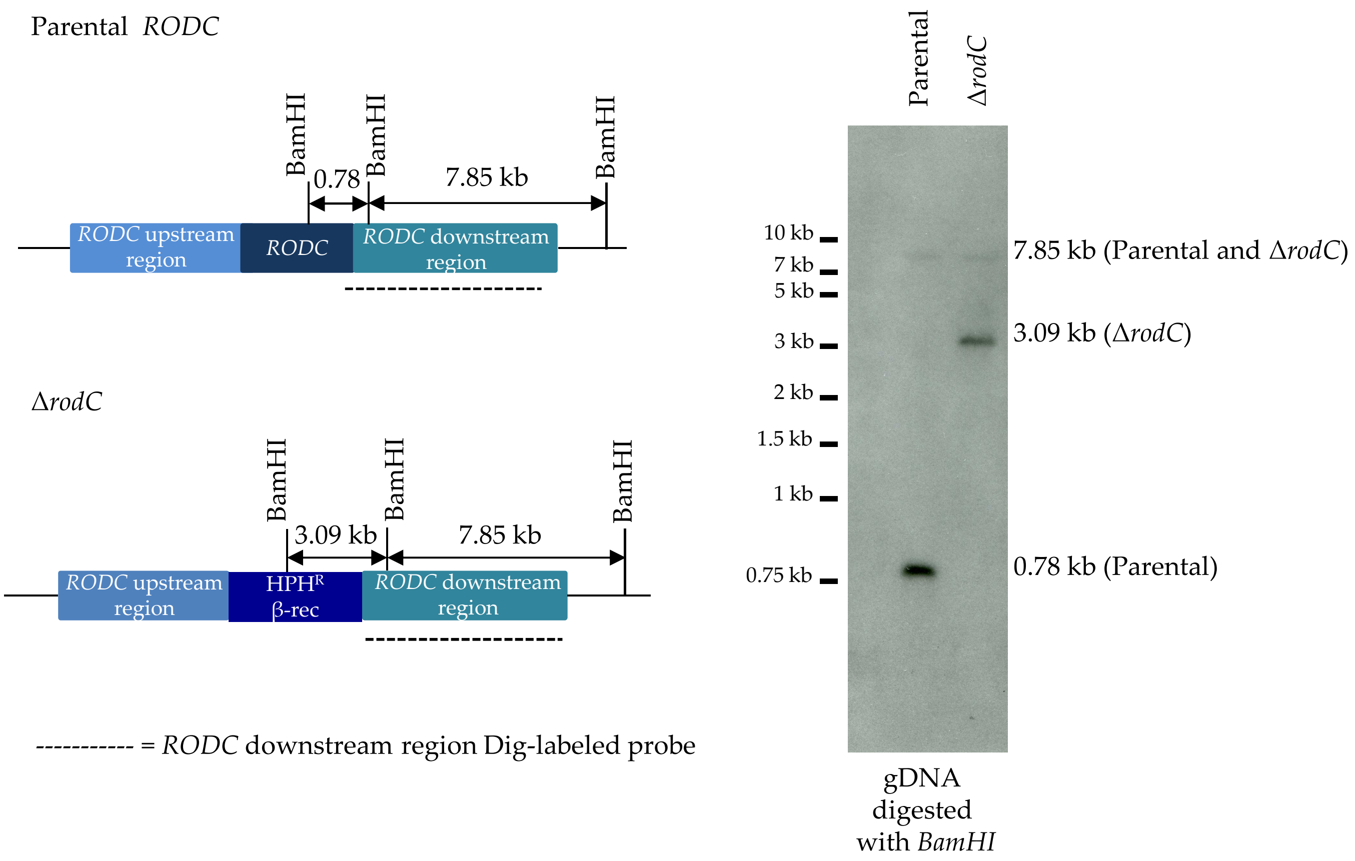

Figure S1C: Southern blot of the *A. fumigatus* Δ*rodC* strain

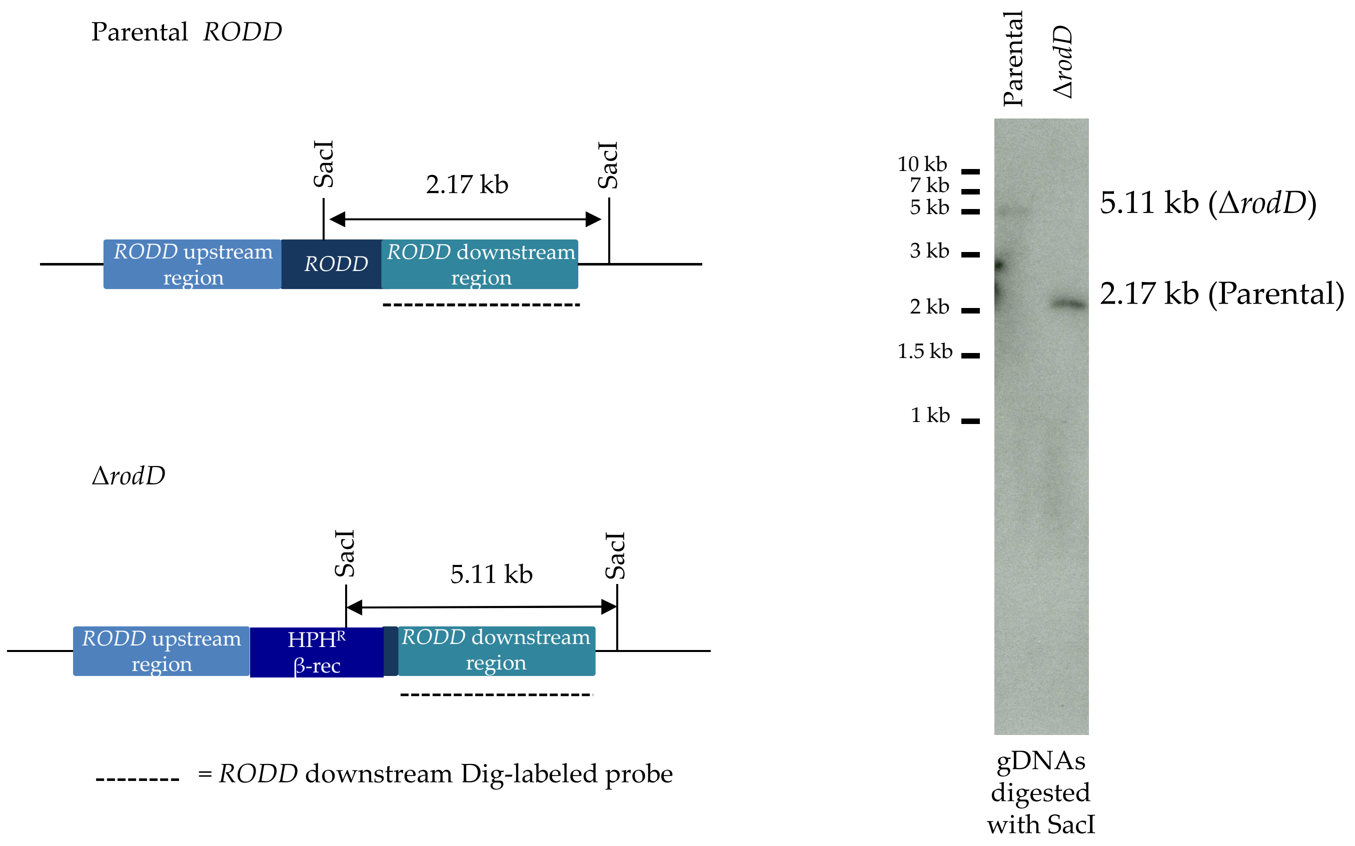

Figure S1D: Southern blot of the *A. fumigatus* Δ*rodD* strain

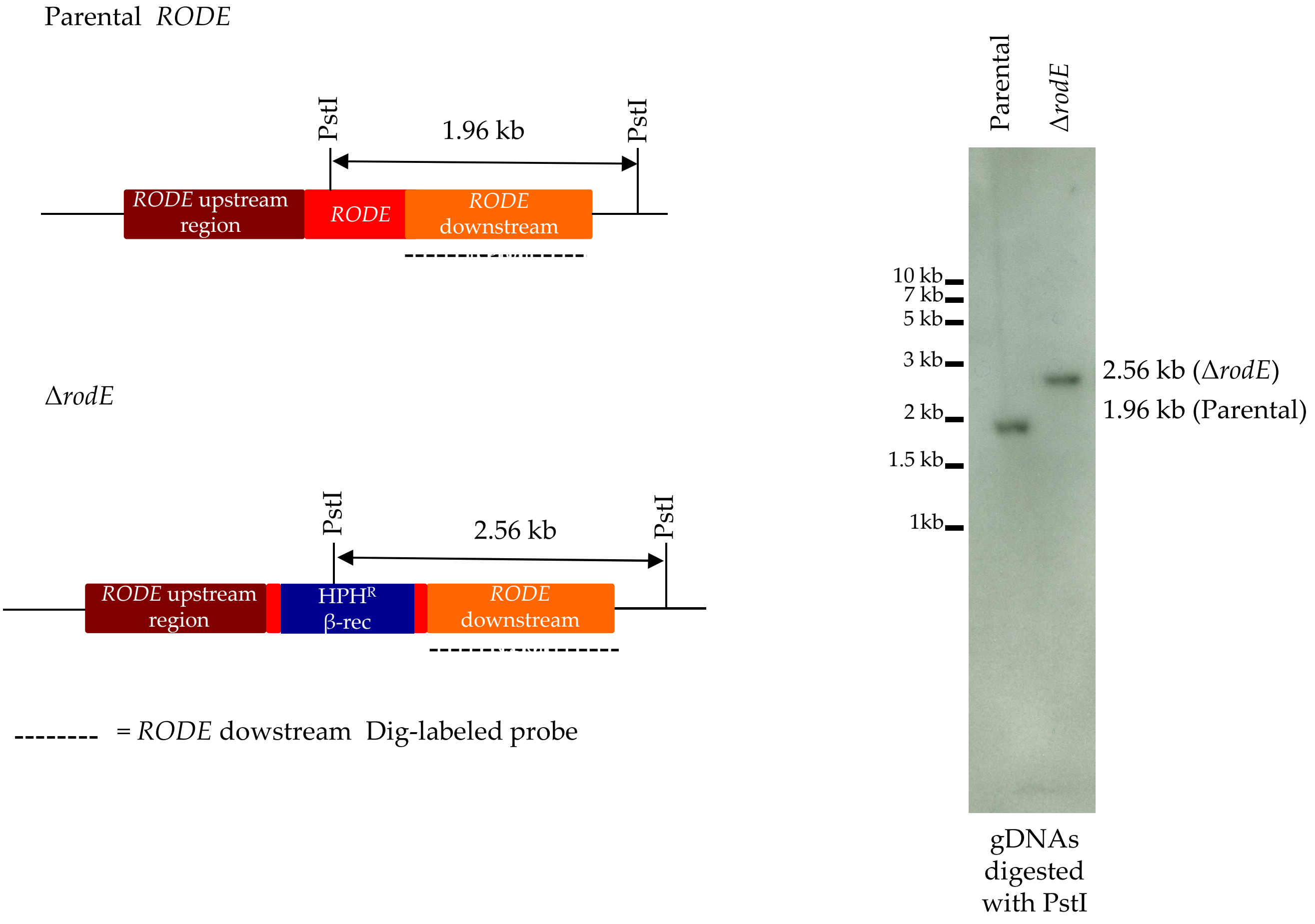

Figure S1E: Southern blot of the *A. fumigatus* Δ*rodE* strain

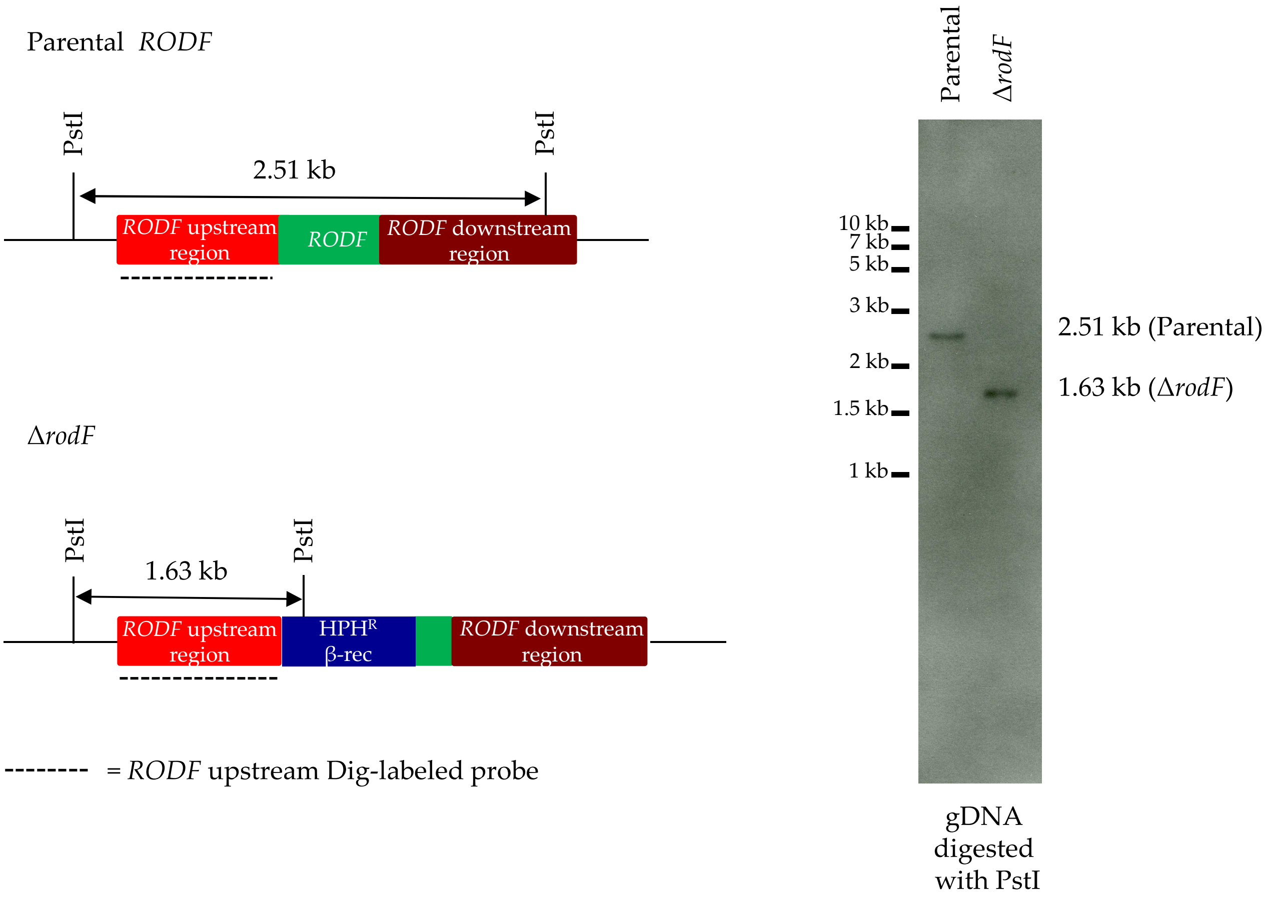

Figure S1F: Southern blot of the *A. fumigatus* Δ*rodF* strain

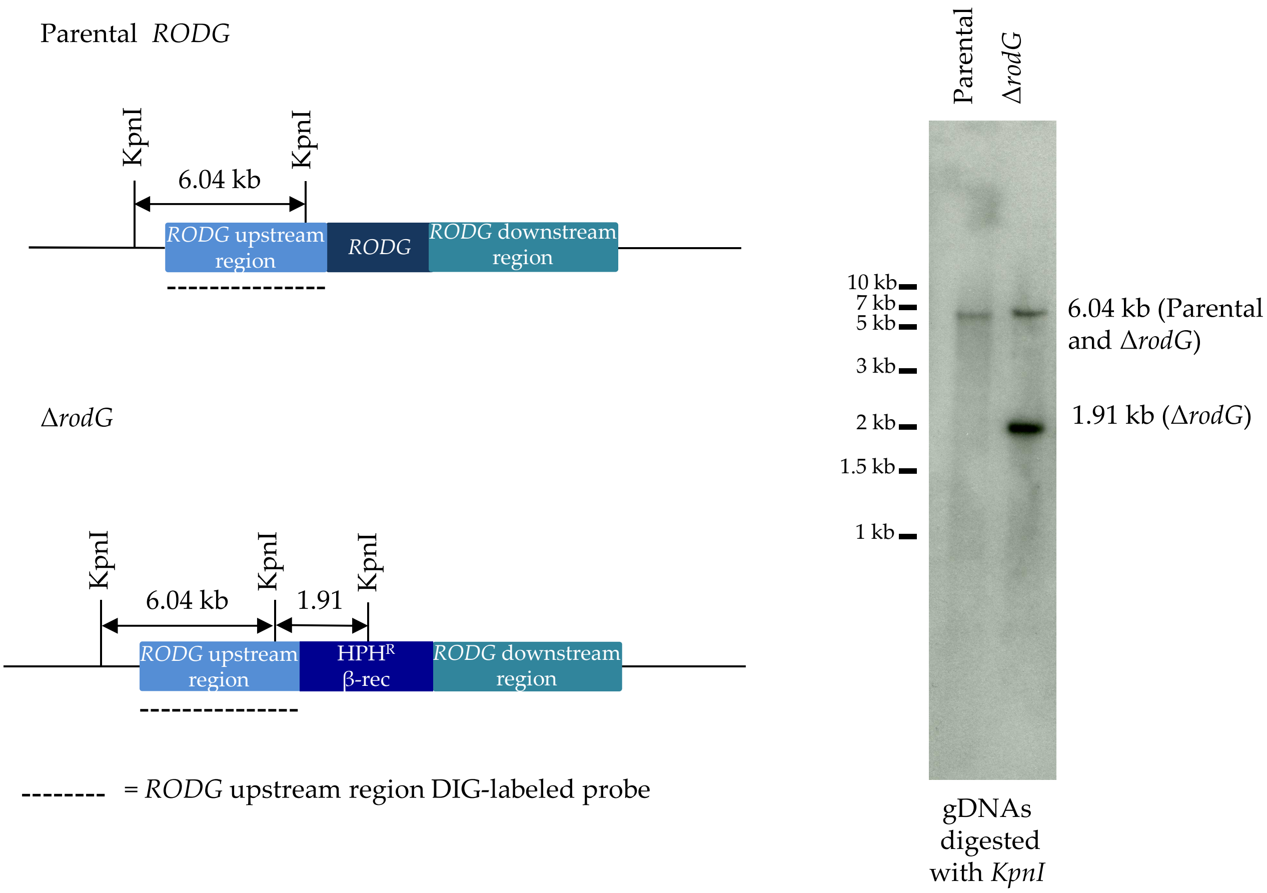

Figure S1G: Southern blot of the *A. fumigatus* Δ*rodG* strain

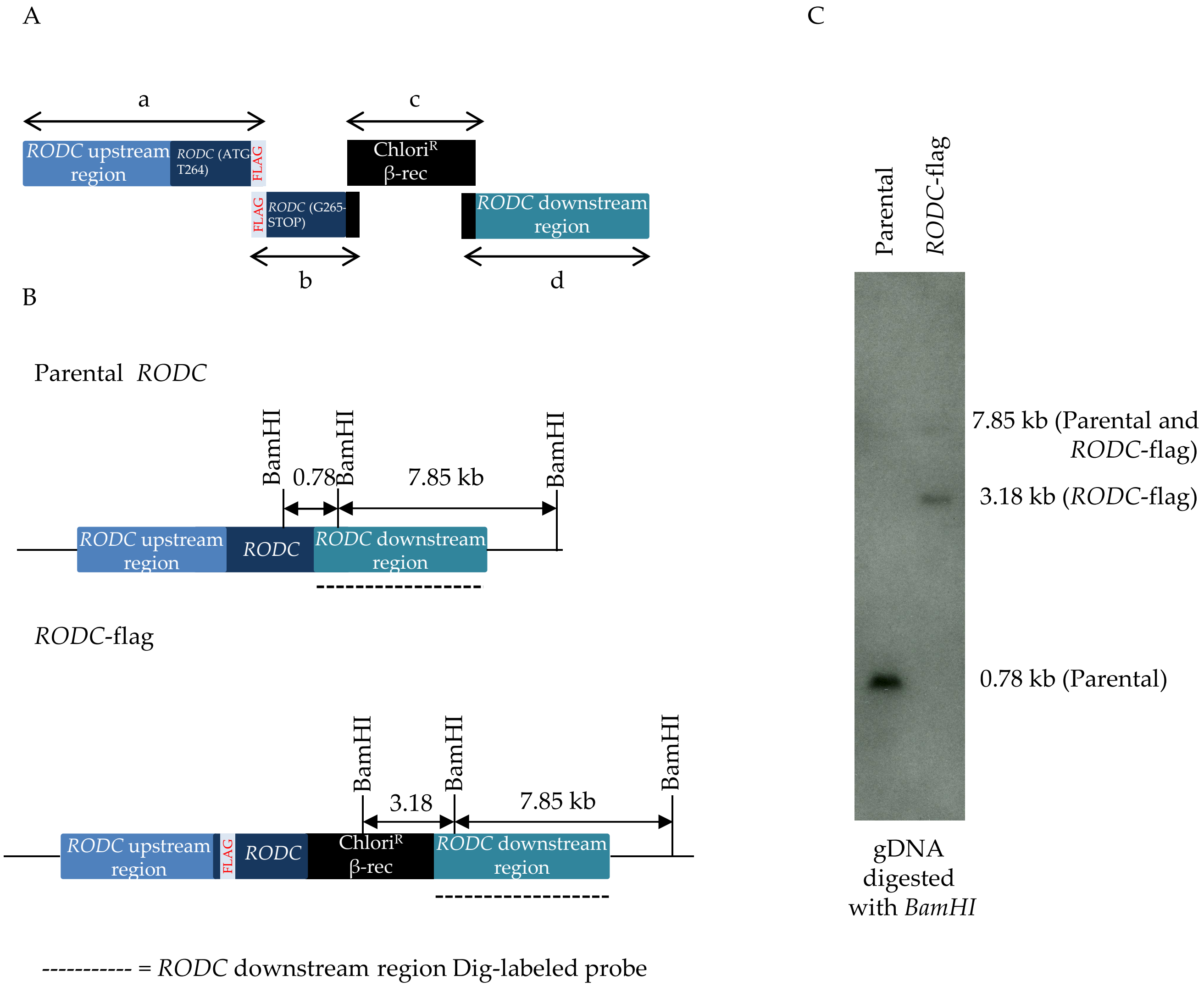

Figure S1H: Construction of the *A. fumigatus* RodC-flag strain. A. Schema of the 4 DNA fragments used for the RodC-flag DNA construct. B. Diagram showing *BamHI* digestion site and Dig labelled probe localization. C. Southern blot analysis. gDNA of parental and RodC-flag strain were digested with *BamHI*, run it on a 0.7% agarose gel and transferred in a nitrocellulose membrane, which was then annealed with the DIG labelled probe containing the downstream border as is shown in the diagram B.

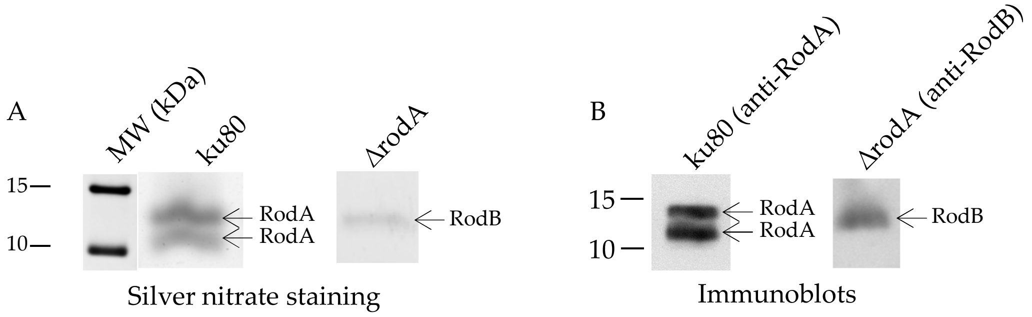

Figure S2 : immunoblotting localization of RodA and RodB using the newly prepared anti-recombinant RodA and RodB antisera on formic acid (for RodA) or TFA-soluble SDS-insoluble extracts (for RodB) of ku80 and Δ*rodA* conidia. Upon extraction, RodA produces two bands corresponding to 14.4 kDa and 12.2 kDa as already described (Aimanianda *et al*. 2009). Because of the similar size of the expected mature protein for RodB (12.8 kDa), RodB was observed in Δ*rodA.* A: SDS-PAGE (15% polyacrylamide) gels were stained with silver nitrate or B: transferred to nitrocellulose and probed with polyclonal anti-RodA (on ku80 extract) or anti-RodB (on Δ*rodA* extract) antibodies.

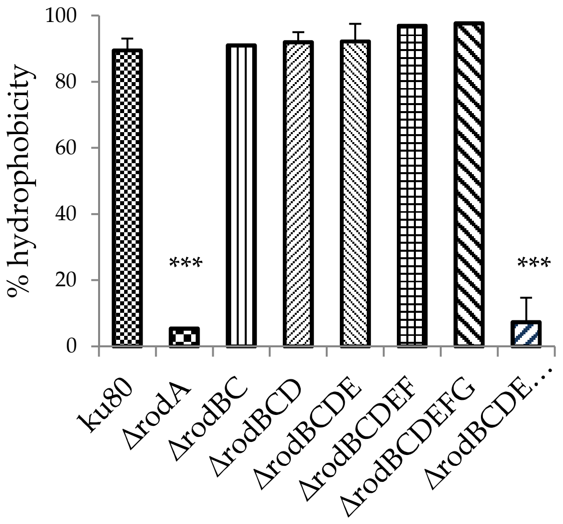

Figure S3 : Hydrophobicity of hydrophobin mutant conidia and parental strain ku80. ***, P < 0.001.

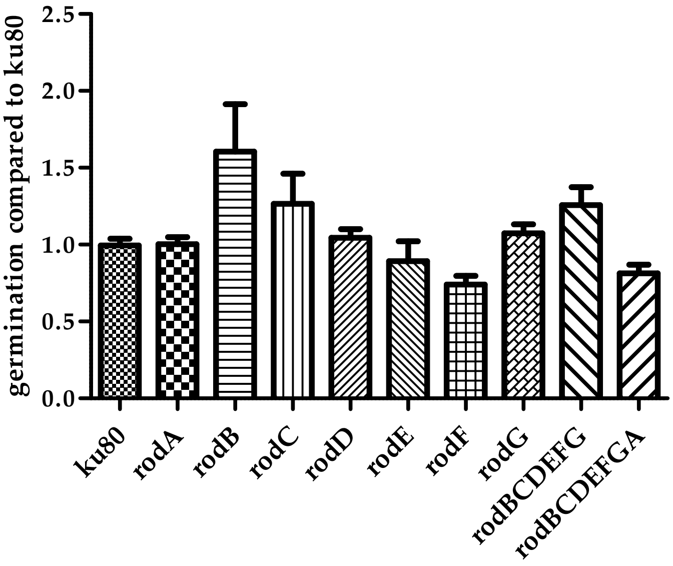

Figure S4 : Germination of hydrophobin mutants in GYE medium after 7 hr at 37°C. Germination rates were normalized to the culture compared with the parental strain ku80, with 1 representing 60 to 75 % germination. Statistical tests were performed with Graph Pad Prism 3.0 and showed non significative difference between the strains.

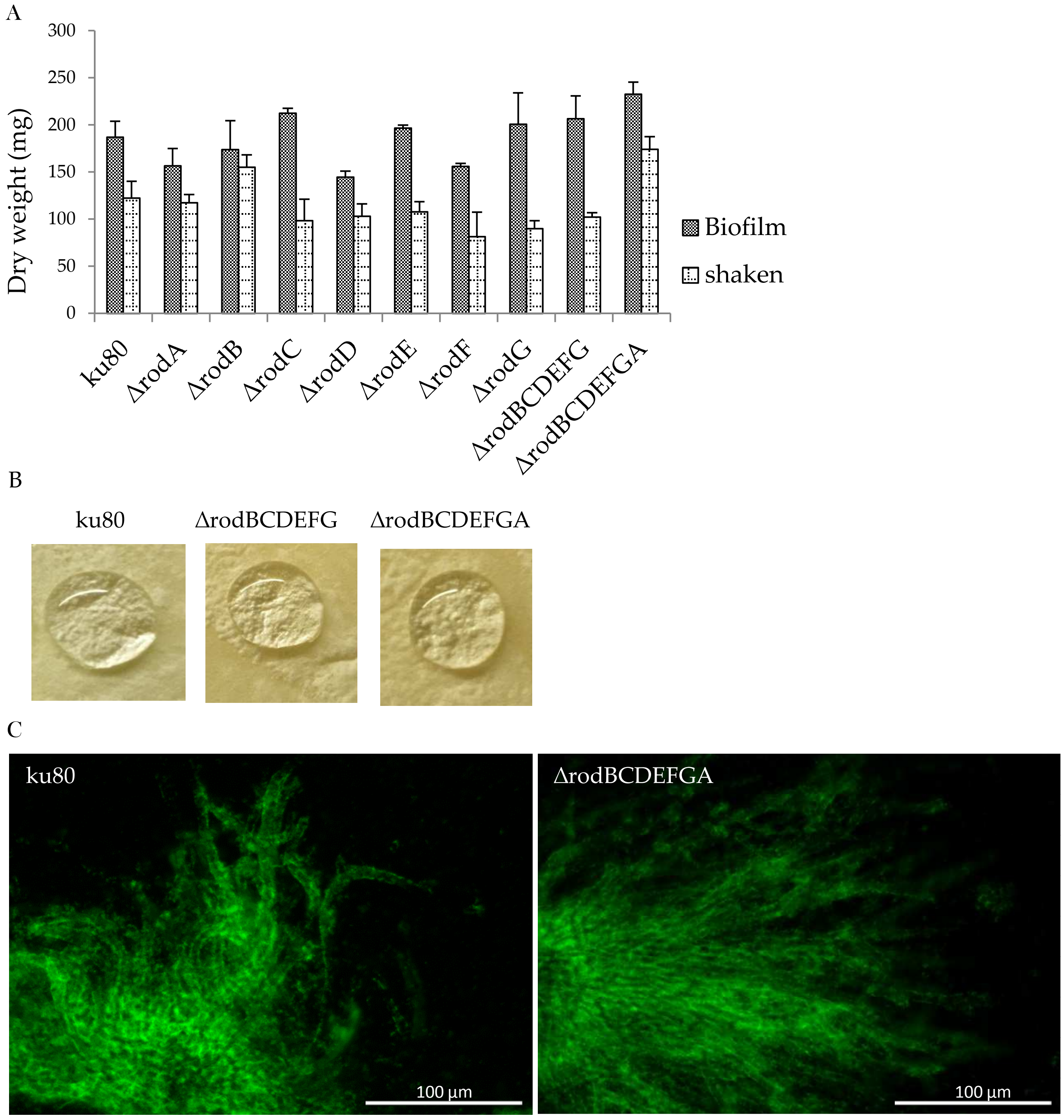

Figure S5 : Characteristics of the mycelium of ku80 and hydrophobin mutants in aerial and static biofilm or submerged and shaken planktonic conditions . A: Biomass (dry weight of the recovered mycelium after 24 hr growth at 30°C), showing similar growth of hydrophobin mutants and ku80; B: hydrophobicity of biofilms observed by placing 10 µL drops of 0.2% SDS in 50 mM EDTA on the surface of the biofilm, showing that the surface of the biofilm of all strains presented the same hydrophobicity (no soaking of the drops into the biofilm); C: binding of *P. aeruginosa* Pa14 (GFP-strain), on ku80 and Δ*rodBCDEFGA* hyphae, showing a similar binding of the bacteria on the hydrophobin mutant and parental strain hyphae.
